# Glucagon-like peptide-1 receptor agonist, liraglutide, attenuated retinal thickening in spontaneously diabetic Torii fatty rats

**DOI:** 10.1186/s12886-022-02413-y

**Published:** 2022-05-06

**Authors:** Kazuho Inoue, Shohei Yamada, Seiko Hoshino, Minoru Watanabe, Kenjiro Kimura, Atsuko Kamijo-Ikemori

**Affiliations:** 1 Department of Anatomy, St. Marianna University School of Medicine, 2-16-1 Sugao, Miyamae-Ku 216-8511 Kanagawa, Japan; 2 Division of Nephrology and Hypertension, Department of Internal Medicine, St. Marianna University School of Medicine, 2-16-1 Sugao, Miyamae-Ku 216-8511 Kanagawa, Japan; 3 Institute for Animal Experimentation, St. Marianna University Graduate School of Medicine, Kanagawa, Japan; 4 JCHO Tokyo Takanawa Hospital, Tokyo, Japan

**Keywords:** Blood Glucose, Chemokine CCL2, Endotherial nitric oxide, Glucagon-like peptide-1 receptor, Liraglutide

## Abstract

**Background:**

This study aims to investigate the effect of the glucagon-like peptide-1 (GLP-1) receptor agonist (GLP-1RA) liraglutide on retinal pathological findings as compared with insulin and hydralazine using an animal model of type 2 diabetes with obesity, hypertension, and hyperlipidemia.

**Methods:**

Male spontaneously diabetic Torii (SDT) fatty rats at 8 weeks of age were randomly assigned to three groups: the liraglutide group (SDT-lira, *n* = 6) received a subcutaneous injection of liraglutide from the age of 8 to 16 weeks, the SDT-ins-hyd group (*n* = 6) was provided both insulin against hyperglycemia and hydralazine against hypertension to match levels of both blood glucose and blood pressure to those of the liraglutide group, and the control group of SDT fatty rats (SDT-vehicle, *n* = 7) and a nondiabetic control group of Sprague–Dawley rats (SD, *n* = 7) were injected with vehicle only. Both eyeballs of all groups were collected at the age of 16 weeks.

**Results:**

Retinal thickness, which was found in the SDT-vehicle group, was significantly prevented to similar levels in both the SDT-lira and SDT-ins-hyd groups. Immunohistological analysis revealed that GLP-1 receptor was not expressed in the retina of all rats. The ocular protein expression of monocyte chemoattractant protein-1, which causes a proinflammatory situation, was significantly upregulated in all SDT fatty rats as compared to SD rats, but the expression levels were similar between all SDT fatty rats. With regard to neovascularization in the eyes, there were no significant differences in protein expressions of vascular endothelial growth factor, CD31, or endothelial nitric oxide synthase in all rats.

**Conclusions:**

The present study indicates that liraglutide prevents retinal thickening, dependent on blood glucose and blood pressure levels in SDT fatty rats without ocular neovascularization. However, the effects did not improve the ocular proinflammatory state.

**Supplementary Information:**

The online version contains supplementary material available at 10.1186/s12886-022-02413-y.

## Background

Diabetic retinopathy (DR) has been reported as occurring in roughly one-third of patients with type 2 diabetes [1]. Patients with type 2 diabetes have various metabolic abnormalities such as hypertension, obesity, and hyperlipidemia, and their disorders are risk factors for the onset and progression of DR. Therefore, appropriate interventions against these disorders beginning in the early phase of type 2 diabetes are indispensable for preventing eyesight loss due to DR [2, 3].

Glucagon-like peptide 1 (GLP-1), which is secreted from L cells in the intestine after a meal, binds to GLP-1 receptor (GLP-1R) expressed in the pancreas and induces insulin secretion, resulting in improvement of blood glucose control. Therefore, GLP-1R agonist (GLP-1RA) is used in patients with type 2 diabetes as an antidiabetic agent. Furthermore, in addition to the pancreas, GLP-1R is expressed in the other organs [4], and GLP-1R is expected to exert pleiotropic effects for organ protections from injury due to diabetes via improvement of various metabolic disorders or activation of another molecular pathways [5].

Recently, some experimental studies have indicated beneficial effects of GLP-1RA against DR in animal models of type 2 diabetes [6, 7]. GLP-1RA may have neuroprotective and vascular-protective actions, resulting in the prevention of progression to advanced DR [8]. In contrast, large clinical cohort studies have reported the possibility that GLP-1RA is related to the progression of DR or the onset of DR [9]. On the other hand, a recent meta-analysis and another clinical study did not show that GLP-1RA contributed to DR aggravation [10, 11]; thus, the influence of GLP-1RA on DR is uncertain in type 2 diabetes. Because the progression of DR may depend on various metabolic disorders in addition to hyperglycemia in patients with type 2 diabetes, we investigated the effect of liraglutide, a GLP-1RA, on DR in a rat model of type 2 diabetes using spontaneously diabetic Torii (SDT) fatty (SDT.Cg-Lepr^fa^/JttJcl) rats, which resemble the human pathophysiology of type 2 diabetes with hypertension, obesity, and hyperlipidemia [12, 13].

## Methods

### Animals

Male SDT fatty rats derived from a Sprague–Dawley (SD) colony were used as a type 2 diabetes model [14]. We previously reported that injection of GLP-1RA decreased the levels of both blood glucose and blood pressure and brought beneficial effects on both kidney and muscle in male SDT fatty rats, independent on blood glucose and blood pressure levels [15, 16]. Rats were subcutaneously injected with GLP-1RA from the age of 8 weeks to 16 weeks [16]. At age of 16 weeks, under general anesthesia using the inhalation anesthetic 2% isoflurane, both eyes were removed after the kidney was isolated [16] to assess the effect of GLP-1RA on the retina for efficient use of experimental animals according to “Reduction” of “3R indicated in Act on Welfare and Management of Animals.” We purchased 5-week-old male SDT fatty rats (*n* = 19) and age-matched control SD rats (*n* = 7) from CLEA Japan (Tokyo, Japan). All rats were housed at the Institute for Animal Experimentation at St. Marianna University School of Medicine under 24°C temperature and a 12-hour light/dark cycle. Rats were allowed free access to usual laboratory chow (CRF-2; Charles River Laboratories Japan, Yokohama, Japan) and water. All procedures performed in studies involving animals were conducted in accordance with the ethical standards of the St. Marianna University School of Medicine institution or practice at which the studies were conducted. The experimental protocol was approved by Animal experiment Committee at Institute for Animal Experimentation, St. Marianna University Graduate School of Medicine (approval No. 1908010 and 2008013).

### Treatments

**Fig. 1:**
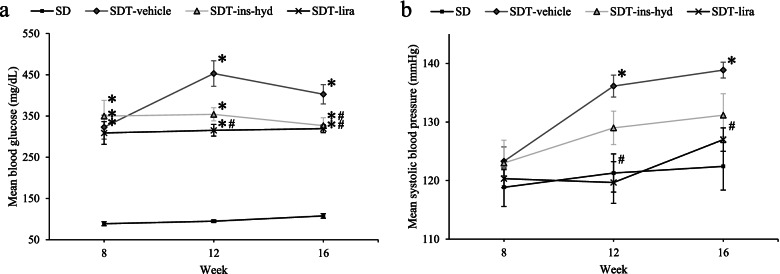
Time-related changes in the blood glucose (**a**) and the systolic blood pressure (**b**) of each group. All data were expressed as mean ± SEM. * *P* < 0.05 versus the SD group; # *P* < 0.05 versus the SDT-vehicle group among the SDT fatty rats. This figure was created based on the results of reference No. 16

Treatments were performed as previously described [16]. Briefly, the 8-week-old SDT fatty rats were randomly divided into three groups. The liraglutide group (liraglutide; *n* = 6) was injected subcutaneously with liraglutide (Novo Nordisk, Bagsværd, Denmark) twice a day for 8 weeks. The daily dosage was increased stepwise from 0.2 mg/kg, which is as follows: 0.2 mg/kg for 1 week, 0.4 mg/kg for 1 week, and 0.6 mg/kg for 6 weeks. Another treatment group (insulin and hydralazine; *n* = 6) was provided both insulin (Humalin N; Eli Lilly, Indianapolis, IN, USA) against hyperglycemia and hydralazine (Sigma-Aldrich Corporation, St. Louis, MO, USA) against hypertension to match levels of both blood glucose and blood pressure with those of the liraglutide group. The daily dosage of insulin was increased stepwise every week from 2U/day to 16U/day. Hydrazine was administered by drinking water to which it had been added at a dosage of 0.5 mg/kg/day. The control group of SDT fatty rats (*n* = 7) and the nondiabetic control group of SD rats (*n* = 7) were injected with vehicle only. The previous reports showed that the blood glucose levels of the SD rats, SDT-vehicle, SDT-ins-hyd and SDT-lira at 8 weeks of age were as follows; 88.86 ± 4.94, 323.14 ± 29.11, 350.00 ± 37.94 and 309.00 ± 27.62, respectively, and at 16 weeks of age were as follows; 107.71 ± 5.53, 402.86 ± 23.39, 326.83 ± 18.87 and 319.40 ± 9.13, respectively (the data expressed as means ± standard error of mean (SEM)) (Fig. 1a) [16]. On the other hand, the systolic blood pressure values of SD rats, SDT-vehicle, SDT-ins-hyd, SDT-lira at 8 weeks of age were as follows; 118.86 ± 3.28, 123.29 ± 2.47, 123.00 ± 3.90, 120.33 ± 1.54, and at 16 weeks of age were as follows; 122.43 ± 4.06, 138.86 ± 1.35, 131.17 ± 3.66, 127.00 ± 2.00, respectively (the data expressed as means ± SEM) (Fig. 1b) [16]. Figure 1 was created based on the results of reference No. 16. The identical levels of both blood glucose and blood pressure were ensured in SDT-ins-hyd and SDT-lira groups.

In both eyes of rats collected at the age of 16 weeks, the right eye was separated immediately, frozen in liquid nitrogen, and stored at − 80 °C until protein expression was assessed. For histological analysis, the left eye was excised and fixed in Super Fix (KY-500, Kurabo, Osaka, Japan), which was diluted three times by phosphate-buffered saline and added to 10% acetic acid for 24 h.

### Measurement of retinal thickness

The fixed left eyes were embedded in paraffin and sectioned to 3-µm thickness. The sections were stained with hematoxylin and eosin (HE) and retinal thickness measured using a ZEISS Axio Imager 2 with ZEN 2 pro imaging software (Carl Zeiss Microscopy, Jena, Germany). Total retinal thickness was defined as the distance between the retinal internal limiting membrane (ILM) and the retinal pigment epithelium. Mean retinal thicknesses were measured at 500, 1000, and 1500 µm from the optic nerve disc according to the previous report [17]. The thicknesses between the ILM and the ganglion cell layer (GCL), the inner plexiform layer (IPL), the inner nuclear layer (INL), the outer plexiform layer, the outer nuclear layer (ONL), and the photoreceptor layer (PL) were also evaluated.

### Immunohistochemical analysis for GLP-1R and CD31

Ocular sections were stained by the standard indirect immunoperoxidase method using primary antibodies against GLP-1R (rabbit monoclonal; 1:1000; abcam, Cambridge, UK) and CD31 (rabbit monoclonal; 1:500; abcam). Antigen retrieval of the sections was performed using a microwave for 15 min in heated Tris–EDTA buffer (pH 9.0). Labeled proteins were visualized using polymeric horseradish peroxidase–conjugated secondary antibodies (ImmPRESS™ polymer detection kit; Vector Laboratories, Burlingame, CA, USA). Peroxidase activity was detected via the diaminobenzidine reaction (Liquid DAB + ; DAKO Japan, Tokyo, Japan), and sections were counterstained with hematoxylin.

To detect the localization of GLP-1R, we used the pancreatic tissue of the SD rats, which was obtained previously, as a positive control for GLP-1R. In addition, as a negative control for GLP-1R in the pancreatic tissue, the pancreatic section was stained without incubating the primary antibody against GLP-1R.

For quantification, CD31-positive areas within 1500 µm of the optic nerve disc in the retinas were measured automatically using an image analyzer (WinRoof version 6.4, Mitani Corporation, Fukui, Japan). The areas positively stained for CD31 were independently measured and expressed as ratios relative to the areas of the entire retinal regions within 1500 µm of the optic nerve disc.

### Assessment of protein expressions for monocyte chemoattractant protein–1

The frozen right eyes were homogenized in T-PER Tissue Protein Extraction Reagent (Thermo Fisher Scientific, Waltham, MA, USA) containing protease inhibitors. The homogenates were centrifuged at 15,000 rpm for 10 min to pellet tissue debris, and supernatant was collected. The protein concentrations were measured by Bradford protein assay (Bio-Rad Laboratories, Hercules, CA, USA). The protein expression of monocyte chemoattractant protein–1 (MCP-1) was measured using an MCP-1 Mouse/Rat Enzyme-Linked Immunosorbent Assay (ELISA) kit (R&D Systems, Minneapolis, MN, USA).

### Assessment of protein expression of vascular endothelial growth factor, CD31, and endothelial nitric oxide synthase

From the extracted protein of the eyes, we measured the protein expression of vascular endothelial growth factor (VEGF) using a VEGF rat ELISA kit (abcam).

For the protein expressions of CD31 and endothelial nitric oxide synthase (eNOS), we performed Western blot analysis. The extracts were separated by NuPAGE 4% to 12% Bis–Tris gels (Invitrogen, Carlsbad, CA, USA) and transferred to a polyvinylidene fluoride membrane. Before the primary antibody reaction, the membranes were cut around molecular weight of each targeted molecule. Primary antibodies against CD31 (1:2000; abcam), eNOS (D9A5L; rabbit monoclonal; 1:1000; Cell Signaling Technology, Danvers, MA, USA), and α-tubulin (rabbit monoclonal; 1:8000; abcam) were used. Immunoreactive bands were detected using ECL Prime Western Blotting Detection Reagent (GE Healthcare, Little Chalfont, UK). The expression levels of all proteins were quantified using ImageJ software (National Institutes of Health, Bethesda, MD, USA) and normalized to that of α tubulin.

### Statistical analysis

All values were expressed as means ± SEM. *P* < 0.05 was considered statistically significant. Following the Kruskal–Wallis test, differences among the four groups were compared using the Mann–Whitney *U* test. All statistical analyses were performed using JMP software version 13.2.1 (SAS Institute, Cary, NC, USA).

## Results

### Evaluation of retinal thickness

**Fig. 2:**
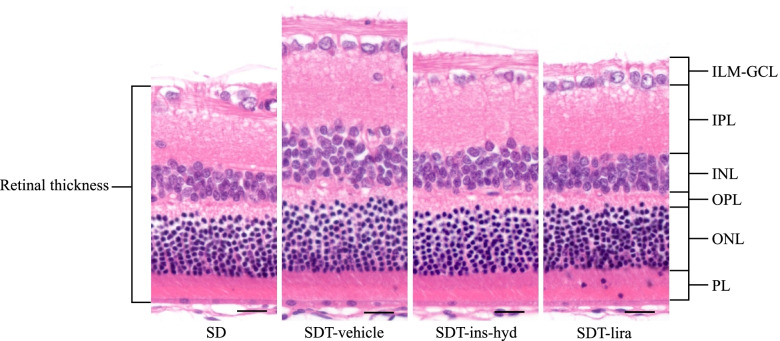
Hematoxylin and eosin staining of the retina at age of 16 weeks in each group. ILM; internal limiting membrane, GCL; ganglion cell layer, IPL; inner plexiform, INL; inner nuclear layer, OPL; outer plexiform layer, ONL; outer nuclear layer, PL; photoreceptor layer. Original magnification: × 200. The scale bars represent 20 µm

**Fig. 3:**
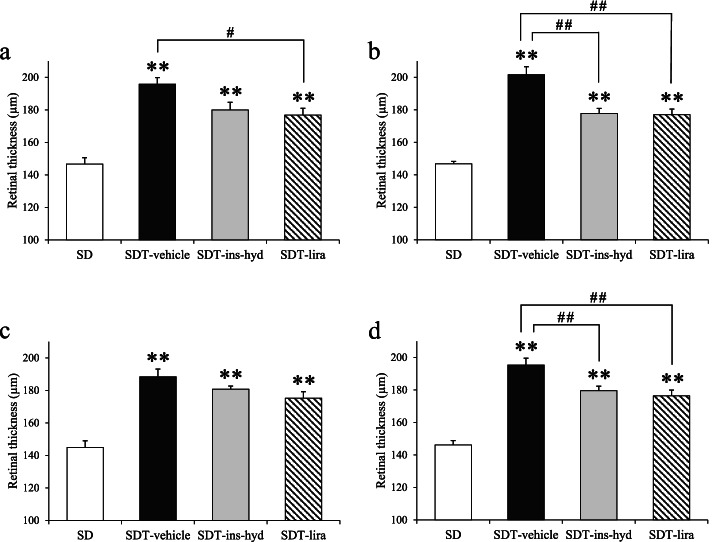
Comparison of retinal thickness at 16 weeks. The retinal thickness at 500 µm from the optic nerve disc (**a**), 1000 µm from the optic nerve disc (**b**), and 1500 µm from the optic nerve disc (**c**). The average of the three measurement points (**d**). All data were expressed as mean ± standard error of the mean (SEM). ^**^ *P* < 0.01 versus the SD group; ^#^ *P* < 0.05, ^##^ *P* < 0.01 versus the SDT-vehicle group among the SDT fatty rats

The retinas in all SDT fatty rats were significantly thicker than those in SD rats in HE-stained ocular tissues (Figs. 2 and 3a–d). In the retina at 500 µm from the optic nerve disc, retinal thickening was significantly suppressed in the SDT-lira group as compared with the SDT-vehicle group (Fig. 3a). In the retina at 1000 µm from the optic nerve disc, retinal thickening was significantly suppressed in the SDT-lira and SDT-ins-hyd groups as compared with the SDT-vehicle group (Fig. 3b). In the retina at 1500 µm from the optic nerve disc, there was no significant difference in retinal thickness between the SDT fatty groups (Fig. 3c). The average of the three measurement points showed that the retinal thickening in the SDT-lira and SDT-ins-hyd groups was significantly prevented as compared with the SDT-vehicle group (Fig. 3d).

**Fig. 4:**
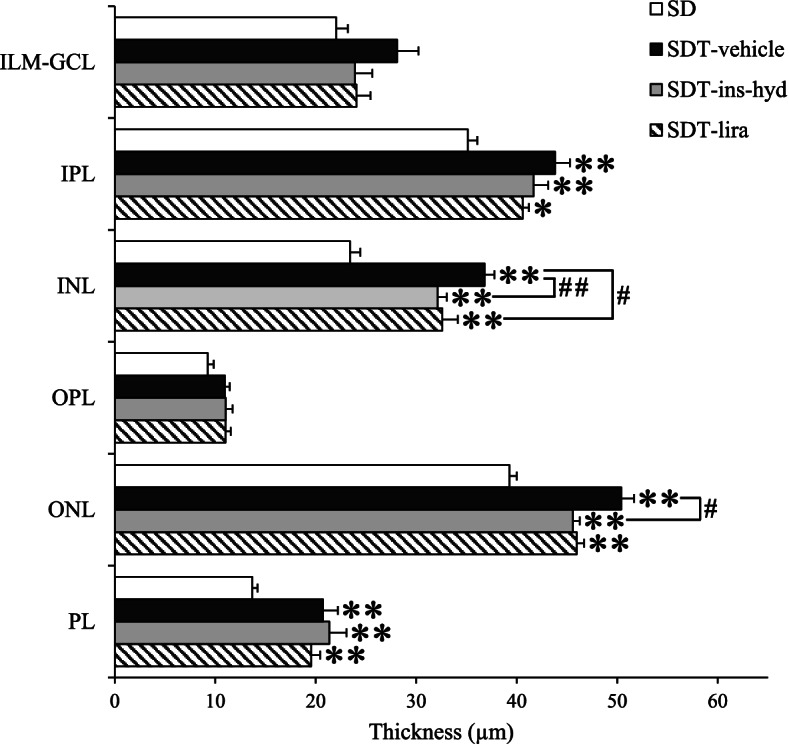
Comparison of the distance between each layer at 16 weeks. The average distance of three measurement points between the ILM and the GCL, the IPL, the INL, the OPL, the ONL, and the PL. All data were expressed as mean ± SEM. ^*^ *P* < 0.05, ^**^ *P* < 0.01 versus the SD group; ^#^ *P* < 0.05, ^##^ *P* < 0.01 versus the SDT-vehicle group among the SDT fatty rats

The retinal layers of IPL, INL, ONL, and PL were significantly thicker in all SDT fatty rats than in SD rats. The layers of INL in the SDT-lira and SDT-ins-hyd groups were significantly thinner than the layer in the SDT-vehicle group. The layer of ONL was significantly thinner in the SDT-ins-hyd group as compared with the SDT-vehicle group (Fig. 4).

### Localization of GLP-1R in retina

**Fig. 5:**
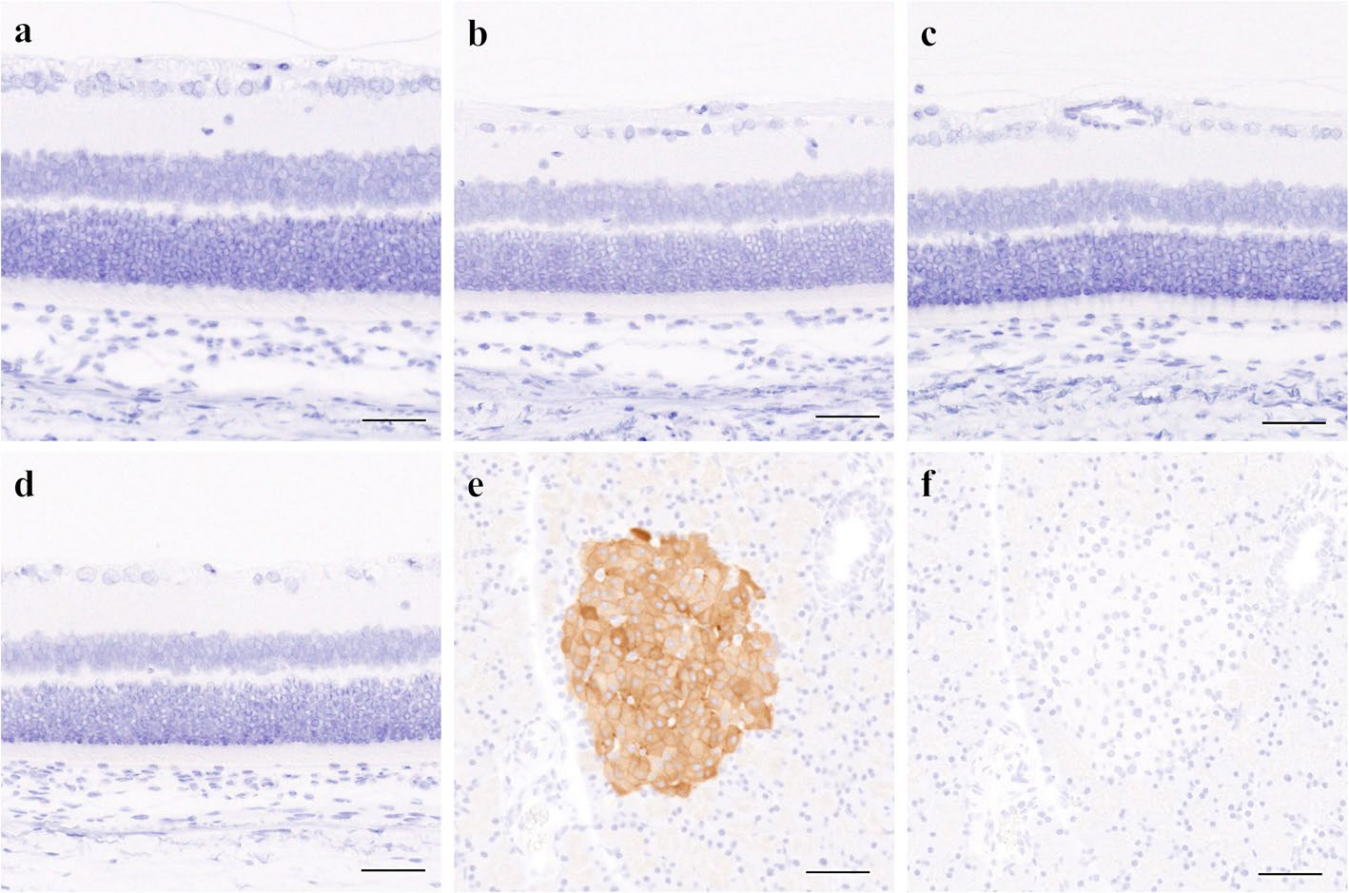
Immunohistological staining using a primary antibody against GLP-1R in retinas of each SD (**a**) and SDT fatty rat (**b**), pancreatic tissue of SD rat as positive control (**c**), and pancreatic tissue of SD rat as negative control stained without incubation of the primary antibody against GLP-1R (**d**). The positive areas of GLP-1R in the retinas were not observed in the SDT-vehicle (**a**), SDT-ins-hyd (**b**), SDT-lira (**c**) and SD (**d**) although that of GLP-1R was confirmed in pancreatic islets (**e**). The negative control of the pancreatic tissue did not show the positive area (**f**). Original magnification: × 200. The scale bars represent 50 µm

Immunohistological analysis showed that the positive area of GLP-1R was not observed in the retinas of all SDT fatty rats (Fig. 5a–c) or SD rats (Fig. 5d), although that of GLP-1R was confirmed in pancreatic islets (Fig. 5e). The positive area was not observed in the pancreatic section stained without incubation of primary antibody against GLP-1R (Fig. 5f).

### Evaluation of MCP-1 protein expression

**Fig. 6:**
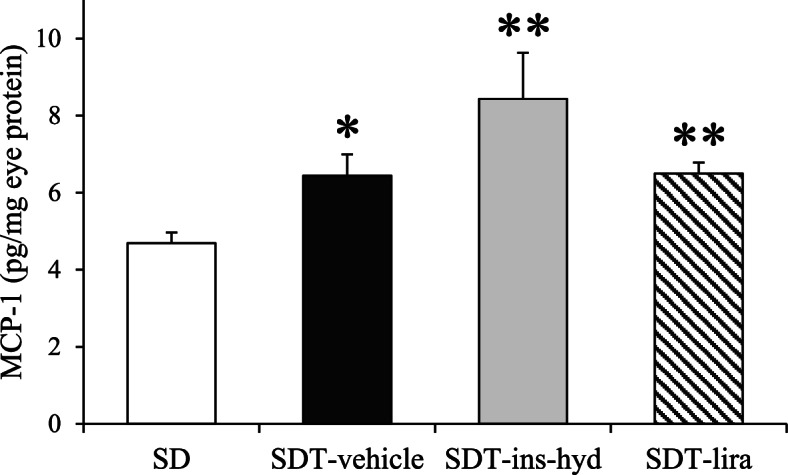
Evaluation of monocyte chemoattractant protein-1 (MCP-1) protein expression in the eyes of each group. All data were expressed as mean ± SEM. ^*^P < 0.05, ^**^ *P* < 0.01 versus the SD group

To evaluate the effects of the treatments on ocular inflammation, we evaluated the protein expression of MCP-1. The expression of MCP-1 was significantly upregulated in the eyes of all SDT fatty rats as compared with those of SD rats, but the expression levels were similar between all SDT fatty rats (Fig. 6).

### Evaluation of VEGF, CD31, and eNOS protein expressions

**Fig. 7:**
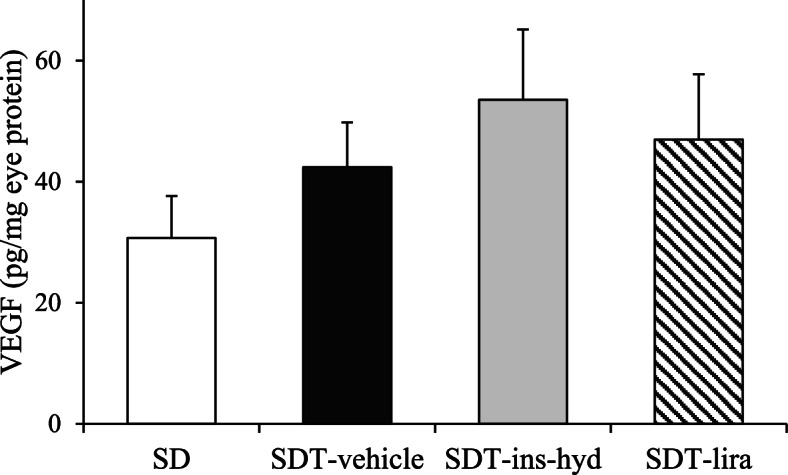
Evaluation of vascular endothelial growth factor (VEGF) protein expression in the eyes of each group. All data were expressed as mean ± SEM

**Fig. 8:**
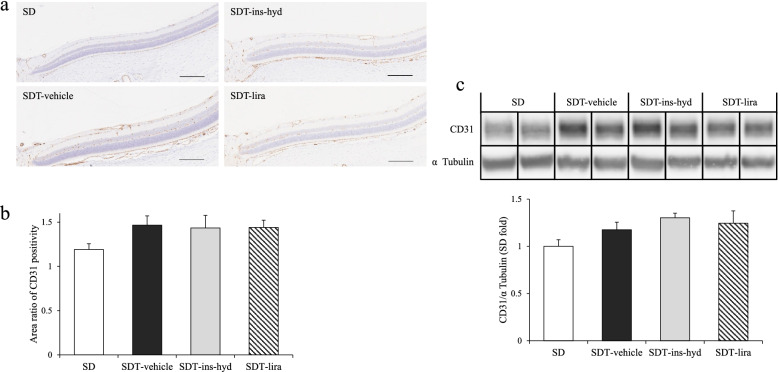
Immunohistological staining using antibodies against CD31 (**a**) and quantitative assessment of CD31 positive areas within 1500 µm of the optic nerve disc (**b**). Original magnification: × 200. The scale bars represent 200 µm. Western blot analysis of eye proteins. Eye protein expressions of CD31 (**c**). Samples from the same experiment were processed in parallel for SDS polyacrylamide gel electrophoresis (SDS-PAGE) and western blotting using different gels and membranes, and the image data obtained were cropped. Entire images of western blotting are shown in online supplementary resource Figure S1 (a and b). All data were expressed as mean ± SEM

**Fig. 9:**
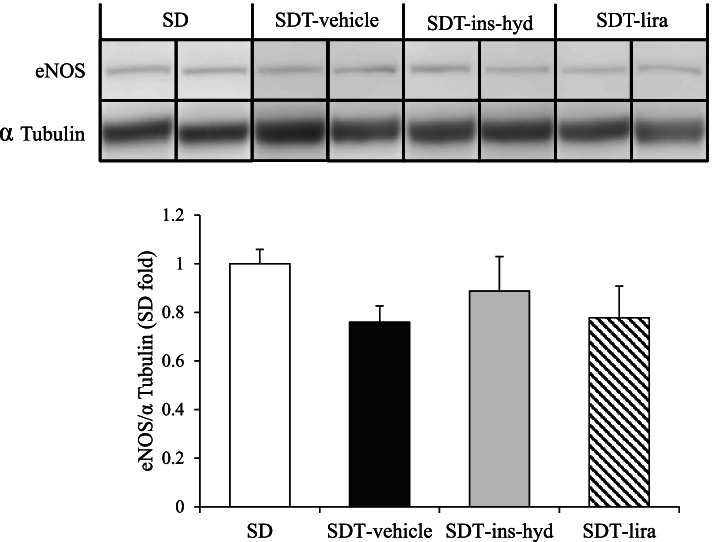
Western blot analysis of eye proteins. Eye protein expressions of endothelial nitric oxide synthase (eNOS). Samples from the same experiment were processed in parallel for SDS-PAGE and western blotting using different gels and membranes, and the image data obtained were cropped. Entire images of western blotting are shown in online supplementary resource Figure S1 (c and d). All data were expressed as mean ± SEM

To reveal the influence of treatments on neovascularization, we evaluated the protein expressions of VEGF, CD31, and eNOS. There were no significant differences in VEGF levels in the proteins extracted from the eyes between all groups (Fig. 7). On immunohistological analysis, the expressions of CD31, which is a marker of endothelial cell, were observed in blood vessel walls in the retinas of all rats (Fig. 8a), and the degrees of the CD31-positive areas were found to be similar in all rats (Fig. 8b). Furthermore, Western blot analysis showed that the expression levels of CD 31 (Fig. 8c) and eNOS (Fig. 9) in the eyes were not significantly different in all groups.

## Discussion

The present study shows that liraglutide suppressed retinal thickening with a significant decrease in the thickness of INL to the same levels as that of insulin–hydralazine treatment in the retina, but it did not prevent the upregulation of ocular inflammatory cytokines in SDT fatty rats without ocular neovascularization. Furhermore, the localization of GLP-1R was not observed in the retinal tissue of SDT fatty rats. The beneficial effects of liraglutide on retinal thickening might be due to both blood glucose– and blood pressure–lowering effects in the SDT fatty rats because there were no significant differences between the two kinds of treatment groups. However, these effects did not extend to the prevention of the proinflammatory situation in the eyes.

Macular edema and proliferative DR are the principal causes of vision loss due to DR. Because macula retina does not exist in rodents, we evaluated retinal thickening, which is the pathological finding of retinal edema, in the present study. On the other hand, because the ocular expressions of VEGF, CD31, and eNOS did not change in all rats, the effects of the treatments on proliferative DR due to retinal neovascularization were not evaluated.

Although advanced diabetic macular edema is treated by laser photocoagulation and intravitreal injections of corticosteroids or anti-VEGF agents [18, 19], there is a need to develop relevant strategies from the early stage of DR to obtain more efficient outcomes against macular edema [20]. In the clinical investigations focused on the associations between DR involving macular edema and systemic diseases, hypertension in addition to hyperglycemia was found to be a crucial risk factor developing DR [3, 21, 22]. Because liraglutide exerted hypotensive effects in SDT fatty rats in our previous study [16], the utility of liraglutide in type 2 diabetes might be useful for preventing the onset or progression of diabetic macular edema.

In diabetes, the accumulation of extra substances in the intracellular and/or extracellular spaces of the retina induces retinal edema, and intracellular edema causes cytotoxic action [23]. Because INL and ONL contain various neuronal cell bodies of retinal cells, thinning of the INL by both liraglutide and insulin–hydralazine treatment and that of ONL by insulin–hydralazine treatment evokes a decrease in intracellular edema via the attenuation of hyperglycemia and hypertension. Such thinning effects may lead to the protection of the retinal cells in those layers. However, because the ocular production of proinflammatory cytokines was not attenuated by these treatments, whether the treatments are able to prevent ocular dysfunction, such as low vision or visual loss, remains uncertain.

Because we previously found that GLP-1R was expressed in the vascular walls of both kidney and muscle [16, 24], we presumed the expression of GLP-1R in the vascular walls of the retina. However, contrary to our expectations, the localization of GLP-1R was not found in the retinas of either SD or SDT fatty rats, and we did not observe a more effective response of liraglutide on the retina beyond blood glucose– and blood pressure–lowing effects induced by insulin–hydralazine treatment. Other groups reported that GLP-1R was expressed primarily in the GCL of human retinas and in the retina of type 2 diabetic model mice (C57BL/KsJ-db/db mice), and they also showed that retinal neuroprotective effects were induced by the activation of GLP-1R using a topical administration of native GLP-1 apart from liraglutide, independent of blood glucose levels [6]. On the other hand, a clinical meta-analysis showed that GLP-1RA did not possess specific effects on the retarding of DR [10], apart from other diabetic complications, such as cardiovascular disease [9] or nephropathy [25], which did not support the protective action due to direct GLP-1R activation in the experimental study. In our study, liraglutide did not suppress the production of proinflammatory cytokines in the eyes, although GLP-1RA was reported to exert an anti-inflammatory action in the retina [7]. To clarify the effect of GLP-1RA in DR, further studies are needed, and a clinical study (the Focus trial) is ongoing (NCT 03811561){ ClinicalTrials.gov NCT03811561. A Research Study to Look at How Semaglutide Compared to Placebo Affects Diabetic Eye Disease in People With Type 2 Diabetes (FOCUS). Available at: https://clinicaltrials.gov/ct2/show/NCT03811561 (Accessed 2 June 2020).]}.

This result may be limited by several factors. First, we used the SDT fatty rats in the present study because the SDT fatty rats had various metabolic abnormalities like the patients with type 2 diabetes [26] and therefore, we considered that ocular pathophysiology might be provoked by the same mechanism as the type 2 diabetic patients. On the other hand, while the expression of GLP-1R has been reported in each mouse and human retina, our examination did not show its expression in the rat retina. Although there may be the difference in the GLP-1R expression due to species differences and our results cannot be extrapolated to the type 2 diabetic patients, our results showed the systemic effective action of liraglutide on the retina. Second, other previous study reported the marked retinal thickening and retinal folds in the SDT fatty rats at 24 weeks of age. However, in our previous study, one SDT fatty rat died at around 24 weeks of age due to malnutrition due to the long-term impairment of glucose utilization [27]. Therefore, in the present study, we focused on the early phase of type 2 diabetes in 16-week-old SDT fatty rats. Third, retinal edema due to increase in vascular permeability leads to retinal thickening, but the vascular permeability was not assessed unfortunately in the present study. Because the retinal thickening is the most useful finding for diagnosis of retinal edema in clinical practice, liraglutide may improve retinal edema. Finally, we used proteins extracted from whole eyes, but not only retinas, because we did not have the technology to isolate retinas. Regarding the CD31 expression for evaluation of neovascularization, we evaluated the expression in the retina by immunostaining in addition to western blotting, and the results of the immunostaining supported those of western blotting. Therefore, changes in the expressions of some molecules in ocular protein were considered to reflect their changes in the retina, but this point is needed to be evaluated in detail in the further study.

## Conclusion

Liraglutide prevented retinal thickening, depending on blood glucose and blood pressure levels, in SDT fatty rats without ocular neovascularization. However, liraglutide did not improve the proinflammatory state in the eyes. Further study is needed to reveal the effects of GLP-1RA on decreasing the sight-threatening risk in type 2 diabetes.

## Supplementary Information

**Additional file 1: Figure S1. **Western blot analysis of eye CD31 (**a**) and α-Tubulin on the same membrane (**b**) in each group. Western blot analysis of eye eNOS (**c**), and α-Tubulin on the same membrane (**d**) in each group. The red lines represent the edge of each cut membrane.

## Data Availability

The data used to support the findings of this study are included within the article.

## Abbreviations

ELISA: Enzyme-Linked Immunosorbent Assay
GCL: Ganglion cell layer
HE: Hematoxylin and eosin
ILM: Internal limiting membrane
INL: Inner nuclear layer
IPL: Inner plexiform layer
ONL: Outer nuclear layer
PL: Photoreceptor layer
SDT: Spontaneously diabetic Torii
VEGF: Vascular endothelial growth factor
