# Supplementary material for: Glucagon-like peptide-1 receptor agonist, liraglutide, attenuated retinal thickening in spontaneously diabetic Torii fatty rats
Source: BMC Ophthalmol. 2022 May 6;22:206. doi: 10.1186/s12886-022-02413-y (PMC9074190; doi:10.1186/s12886-022-02413-y)
Supplement: Supplementary file 1 — Additional file 1: Figure S1. Western blot analysis of eye CD31 (a) and α-Tubulin on the same membrane (b) in each group. Western blot analysis of eye eNOS (c), and α-Tubulin on the same membrane (d) in each group. The red lines represent the edge of each cut membrane. [file 12886_2022_2413_MOESM1_ESM.docx]

**Glucagon-like peptide-1 receptor agonist, liraglutide, attenuated retinal thickening in spontaneously diabetic Torii fatty rats**

Kazuho Inoue^1^, Shohei Yamada^2^, Seiko Hoshino^1^, Minoru Watanabe^3^, Kenjiro Kimura^4^, Atsuko Kamijo-Ikemori^1, 2, 3^

1. Department of Anatomy, St. Marianna University School of Medicine, Kanagawa, Japan.

2. Division of Nephrology and Hypertension, Department of Internal Medicine, St. Marianna University School of Medicine, Kanagawa, Japan.

3. Institute for Animal Experimentation, St. Marianna University Graduate School of Medicine, Kanagawa, Japan.

4. JCHO Tokyo Takanawa Hospital, Tokyo, Japan.

**Correspondence should be addressed to:**

Atsuko Kamijo-Ikemori, M.D., Ph.D.

Department of Anatomy, and Division of Nephrology and Hypertension, Department of Internal Medicine, St. Marianna University School of Medicine

2-16-1 Sugao, Miyamae-Ku, Kawasaki 216-8511, Japan

Tel: +81-44-977-8111 ext. 3630, Fax: +81-44-976-7083

E-mail: [a2kamijo@marianna-u.ac.jp](mailto:a2kamijo@marianna-u.ac.jp)

**Additional Figures and Supporting Information**

**Entire images of western blotting**

 **Figure S1**

**b**

**a**

**d**

**c**

**Figure Legends**

**Figure S1** Western blot analysis of eye CD31 (a) and α-Tubulin on the same membrane (b) in each group. Western blot analysis of eye eNOS (c), and α-Tubulin on the same membrane (d) in each group. The red lines represent the edge of each cut membrane.
